# Genetic and Molecular Mechanisms of Hypertrophic Cardiomyopathy

**DOI:** 10.3390/ijms24032522

**Published:** 2023-01-28

**Authors:** Chun Chou, Michael Thomas Chin

**Affiliations:** 1 Department of Medicine, Tufts University School of Medicine, Boston, MA 02111, USA; 2 Molecular Cardiology Research Institute, Tufts Medical Center, Boston, MA 02111, USA

The intention of this Special Issue is to highlight novel approaches and new paradigms for understanding the pathogenesis of hypertrophic cardiomyopathy (HCM). Classically considered a monogenic disorder with an autosomal dominance inheritance pattern, HCM is in fact a common inherited heart disease with heterogenous temporal and phenotypical manifestation. Yet, extensive HCM research has focused on cardiomyocyte contractile machinery in the setting of sarcomere gene mutations, which are only present in a substantial but small fraction of patients with HCM. Disappointingly, the majority of whole-genome sequencing studies in the past have failed to identify additional disease-causing mutations, but have instead uncovered numerous variants of unknown significance. 

The seeming lack of genotype to phenotype correlation and the challenges of interpreting genetic testing are fundamental problems faced by HCM researchers and clinicians in the post-genomic era [1]. In clinical practice, the Mayo HCM Genotype Predictor Score is commonly used to calculate the pre-test probability of having a genetic cause for HCM. Although the algorithm takes into account the features of LV on echocardiogram, the age of disease onset and family history, the overall yield of subsequent genetic testing was only 34%. Thus, the largely unrevealing results from genomic sequencing studies challenge the monogenic origin of HCM and call for collaboration between clinical cardiologists, geneticists and molecular biologists to further define polygenic disease-causing factors. 

Further insight into the complicated molecular pathogenesis of HCM is provided in a review by Chou and Chin [2]. While acknowledging the prevailing cardiomyocyte-centric sarcomere-focused paradigm, the authors also incorporate pathogenic mechanisms beyond sarcomere dysfunction to construct an integrated model of HCM pathogenesis. The review discussed how alterations in calcium handling, autophagy and cellular metabolism contribute to cellular hypertrophy in a cardiomyocyte-intrinsic manner. Additionally, particular focus was placed on the contributions of non-cardiomyocyte cells in driving HCM pathogenesis. Of note, a seminal study by Codden and Chin [3] uncovered substantial alterations in intercellular communication networks in the heart tissues of patients with HCM compared with healthy controls. Single nuclei RNA sequencing analysis followed by in silico interrogation of the cellular interactome demonstrated that many of the dysregulated cell–cell interactions may involve integrin beta 1 and its cognate extracellular matrix ligands. These findings imply pathogenic contributions from non-cardiomyocyte cells, and provide a potential explanation of how sarcomere dysfunction can propagate pathogenic signals among cardiomyocytes and other cell types. 

In addition to genomic and transcriptomic analyses, proteomics and phosphoproteomics studies, which capture post-translational changes that could not otherwise be revealed, may provide further mechanistic insights. A review by Moore and Emili [4] in this issue introduces several key concepts through the use of state-of-the-art mass spectrometry and bioinformatics techniques to assess biological mechanisms. The authors discuss in detail how these techniques can be utilized to understand HCM pathogenesis in cell culture, animal models and human patients with HCM. Beyond proteomics studies on cardiac tissues, Larson et al. [5] profiled plasma proteins in HCM patients prior to or post myectomy surgery using an aptamer-based proteomics platform. The study showed that the plasma proteins involved in VEGF signaling and pro-inflammatory responses were reduced after myectomy, consistent with prior studies implicating the role of angiogenesis and inflammation in HCM pathogenesis. Notably, Larson et al. further identified 25 plasma proteins that distinguish pre- and post-operative states with high statistical confidence. Conceivably, an individualized LVOT obstruction risk score may be derived from the expression of this set of plasma proteins. With additional insights from the obstructive HCM-specific gene expression profile identified by Codden and Chin [3], a refined LVOT-obstruction risk score may be generated to further guide personalized treatment strategies. 

Atrial fibrillation (AF) is one of the most common rhythm disorders in patients with HCM and confers additional risk for other comorbidities, including heart failure and stroke. In this issue, Lim et al. [6] aimed to understand the pathogenic mechanisms of AF in HCM by using a transgenic mouse model of HCM in which a point mutation was introduced to the troponin I allele. The group demonstrated that several properties of the atrial electrical conductance system were already altered early in disease progression, in addition to interstitial fibrosis and inflammation. These defects were further aggravated in aging transgenic mice. Notably, AF was notoriously difficult to induce in mice, and the transgenic mice used by Lim et al. did not develop AF. Nevertheless, this study presented a valuable mouse model with an underlying atrial substrate that may confer a propensity for AF as HCM progresses. 

Thanks to the advancement and affordability of various omics technologies, we are beginning to uncover previously unappreciated cellular contributors, novel inter-cellular communication networks and unexpected alterations in intracellular pathways associated with HCM. Most importantly, these studies may ultimately allow for the identification of patient-specific transcriptomic and proteomic footprints that facilitate Precision Medicine approaches.
